# Associations among S100A4, Sphingosine-1-Phosphate, and Pulmonary Function in Patients with Chronic Obstructive Pulmonary Disease

**DOI:** 10.1155/2022/6041471

**Published:** 2022-02-03

**Authors:** Hou-Ying Qin, Meng-Die Li, Guo-Fang Xie, Wei Cao, De-Xiang Xu, Hui Zhao, Lin Fu

**Affiliations:** ^1^Department of Respiratory and Critical Care Medicine, Second Affiliated Hospital of Anhui Medical University, Hefei 230601, Anhui Province, China; ^2^Department of Toxicology, Anhui Medical University, Hefei 230032, China

## Abstract

**Background:**

S100A4 is a member of the S100 calcium-binding protein family and is increased in patients with chronic obstructive pulmonary disease (COPD). Sphingosine-1-phosphate (S1P) is a naturally occurring bioactive sphingolipid, which regulates the adhesion between the cells and the extracellular matrix and affects cell migration and differentiation. The goal of this study was to analyze the correlations among S100A4, S1P, and pulmonary function among COPD patients.

**Methods:**

All 139 serum samples and 15 lung specimens were collected in COPD patients and control subjects. S100A4 and S1P were detected in two groups. The markers of fibrosis and epithelial-mesenchymal transition (EMT) were measured in the lungs of COPD patients and control subjects.

**Results:**

The protein expression of S100A4 was higher in the lungs and serum of COPD patients than control cases. Additionally, serum S100A4 was inversely associated with pulmonary function among COPD patients. Meanwhile, collagen deposition and EMT nuclear transcription factors were elevated in the lungs of COPD patients. Moreover, the protein expression of S1P was increased in the serum of COPD patients. Serum S1P was gradually increased along with pulmonary function decline in COPD patients. Further correlation analysis revealed that serum S1P was negatively associated with pulmonary function in COPD patients. Furthermore, there was a positive correlation between S1P and S100A4 in COPD patients.

**Conclusions:**

These results provide evidence that the elevation of S100A4 and S1P may be involved in the onset and progression of COPD.

## 1. Introduction

Chronic obstructive pulmonary disease (COPD) is characterized by irreversible airflow limitation and chronic airway inflammation, which evokes a heavy burden on the whole society and individuals [1]. As the aging society approaches, more and more people suffer from COPD and about 3 million people die from COPD every year globally [1]. The number of deaths caused by COPD each year ranks third of all diseases all over the world [2]. Chronic inflammation caused by smoking and harmful particles is an important reason for chronic obstructive pulmonary disease [3]. However, the exact pathogenesis of COPD is unknown. Increasing pieces of evidence have confirmed that pulmonary fibrosis and epithelial-mesenchymal transition (EMT) are involved in the progress of airway restriction and emphysema [4, 5]. However, the exact role of EMT in the occurrence and development of COPD remains to be further elucidated.

S100A4 (fibroblast-specific protein 1) is a member of the S100 calcium-binding protein family. It is generally known that S100A4 exerts central roles in the pathophysiology of tumor metastasis, fibrotic diseases, and autoimmune disorders [6–8]. Recently, it was shown that S100A4 from fibroblasts promotes pulmonary fibrosis [9, 10]. Recent research indicated that S100A4 promotes tumor metastasis and EMT [11]. A few studies have confirmed that S100A4-mediated EMT plays an important role in the occurrence of tumors and nonneoplastic diseases [12–14]. A clinical epidemiological study demonstrated that S100A4 is elevated in the serum of patients with idiopathic pulmonary fibrosis [15]. Sphingosine-1-phosphate (S1P) is a naturally occurring bioactive sphingolipid, which regulates the adhesion between the cells and the extracellular matrix through G protein coupling with S1P receptors, thereby affecting cell migration and differentiation [16]. S1P is phosphorylated by sphingosine kinases (SphK1 and SphK2) that utilize sphingosine during the degradation of plasma membrane glycosphingolipids and sphingomyelin [17]. Additionally, S1P is demonstrated to function as structural components and second messengers [18]. The previous research has revealed that sphingolipid signaling balance exerts a central role in maintaining the homeostasis of bodies [19, 20]. A study has indicated that S1P disorder is related to inflammatory responses and carcinogenesis in different tissues [18]. Animal experiments indicated that pulmonary S1P was elevated in cigarette smoking-induced COPD mice [21]. However, the associations among S100A4, S1P, and pulmonary fibrosis were unknown in COPD patients.

The purpose of this research was to evaluate the associations among S100A4, S1P, and pulmonary fibrosis in COPD patients based on a case-control study. The current research found that S100A4 and S1P were increased in lung tissues and serum samples of COPD patients. The levels of S100A4 and S1P were inversely correlated with pulmonary function in COPD patients. These results have demonstrated that S1P is positively correlated with S100A4 and pulmonary function decline in COPD patients.

## 2. Material and Methods

### 2.1. Reagents

The primary antibodies against *β*-actin (8H10D10) and S100A4 (ab197896) were purchased from Abcam (Cambridge, MA). Antibodies against Twist (10E4E6) and *α*-SMA (ab119952) were obtained from Cell Signaling Technology (Beverly, MA). ECL detection kits were bought from Advansta (CA, USA).

### 2.2. Recruitment of Subjects

All 139 COPD patients were randomly enrolled from the Anhui COPD Cohort (AHCC) study which was a hospital-based prospective cohort study [22–24]. COPD patients meet the diagnostic criteria of the GOLD guidelines. Pulmonary function tests were confirmed in all subjects. COPD was diagnosed according to the criteria of the American Thoracic Society and the Global Initiative for COPD (GOLD). Healthy people with normal pulmonary function were randomly recruited from the physical examination center as the control group. Exclusion criteria are as follows: (1) age < 18 years old; (2) existence of other respiratory diseases, such as bronchial asthma, bronchiectasis, tuberculosis, and lung cancer; (3) combined solid tumors, blood system diseases, immunodeficiency, autoimmune system diseases, pregnancy, major trauma, shock, etc.; (4) history of chemotherapy and radiotherapy in the past 1 month; and (5) use of immunosuppressive agents, steroids, etc., in the past 2 weeks. Every COPD patient was matched with one control subject on the basis of sex and age. All control cases were from the medical examination center in the Second Affiliated Hospital of Anhui Medical University. Blood samples of COPD patients were collected on the morning of the second day after hospitalization. The serum samples were stored in the refrigerator at -87°C after centrifugation [25, 26]. All human lung tissue specimens were collected during surgery. We obtained 15 lung tissues from COPD patients and 15 paracancerous tissues from lung cancer patients without other pulmonary diseases. The lung tissues were removed farther than 5 centimeters from pulmonary focus [27, 28]. This study complies with medical ethics standards and has been approved by the Ethics Committee of Anhui Medical University (LLSC20210822).

### 2.3. Western Blotting

RIPA buffer and PMSF were added to lung tissue to prepare the lung homogenate. All protein samples were separated on a 15% SDS-PAGE gel, and then the samples were transferred to the PVDF membrane. The membrane was washed three times with phosphate-buffered saline after being blocked in the 5% skim milk for 1.5 h at room temperature. The membrane was incubated with different primary antibodies overnight at 4°C in the refrigerator overnight. After being washed three times with phosphate-buffered saline, the secondary antibodies whose source derived from the primary antibodies were continued to incubate for 2 h at 37°C. An ECL kit was used for chemiluminescence detection as previously described [29, 30]. Densitometric analysis was carried out through ImageJ software.

### 2.4. Enzyme-Linked Immunosorbent Assay (ELISA)

S1P ELISA kits were purchased from Wuhan Colorful Gene Biological Technology Co., Ltd. S100A4 ELISA kits were bought from Cusabio, Wuhan, China (https://www.cusabio.com/). The levels of S100A4 and S1P were measured using ELISA according to the manufacturer's protocol [31, 32].

### 2.5. Immunohistochemistry (IHC)

After lung tissue dewaxing, hydration, antigen retrieval, and serum blocking, slides were incubated with primary antibodies *α*-SMA (1 : 300) and Twist (1 : 300) for 36 h at 4°C and 2 h at room temperature. Then, the pulmonary slides were washed in phosphate-buffered saline three times. Next, the pulmonary slides were incubated with the secondary antibodies for 45 min at room temperature. Pulmonary slides were counterstained with PBS after being stained with DAB and hematoxylin. Finally, *α*-SMA-positive cells and Twist-positive nuclei were observed and calculated under a light microscope after dehydration and sealing [33, 34].

### 2.6. Masson's Staining

The paraffin sections were dip-stained in a mordant solution and then stained with lapis lazuli blue and Mayer's hematoxylin. Pulmonary slides were stained with ponceau magenta dye after being differentiated with acid alcohol differentiation solution. Slides were washed with tap water and distilled water in turn and then treated with phosphomolybdic acid solution and aniline blue. Finally, the slides were rinsed, dehydrated, and observed [35].

### 2.7. Statistical Analysis

Statistical analysis was carried out using SPSS version 19.0 software (SPSS Inc., Chicago, IL, USA). All continuous variables were expressed with mean and median. All categorical variables were shown using frequencies and percentages. The difference of continuous variables was evaluated via the *t*-test or the Mann-Whitney *U* test between two groups. All categorical variables were compared using the chi-squared test between two groups. Correlation analysis was accessed through linear regression among COPD patients. *P* < 0.05 was considered statistically significant.

## 3. Results

### 3.1. Demographic Data and Clinical Characteristics

Demographic data and clinical characteristics are presented in Table 1. One hundred thirty-nine COPD patients (74.1% males) and 139 control subjects (71.9% males) were recruited in this study. The mean age was 73.84 and 72.35 years old in COPD patients and control subjects, respectively. The ex-smoker number was more in COPD patients (75.3%) than in control cases (30.2%). The number of white blood cells (WBCs) and neutrophils was increased, and the count of lymphocytes was decreased in patients with COPD. In addition, the levels of C-reactive protein (CRP) and interleukin-6 (IL-6) were obviously higher in COPD patients compared with control subjects. Moreover, the demographic data and clinical characteristics were further analyzed between COPD patients and control cases based on gender. As shown in Supplemental Table 1, the number of ex-smokers was more in male cases than in female cases. In addition, there was no difference in WBCs, neutrophils, lymphocytes, eosinophils, monocytes, and basophils in COPD patients with different genders. Pulmonary function analysis revealed that FEV1% and FEV1/FVC% were increased in female COPD patients than in male patients. On the contrary, FVC was high in male COPD patients.

### 3.2. The Levels of S100A4 between COPD Patients and Control Cases

**Figure 1:**
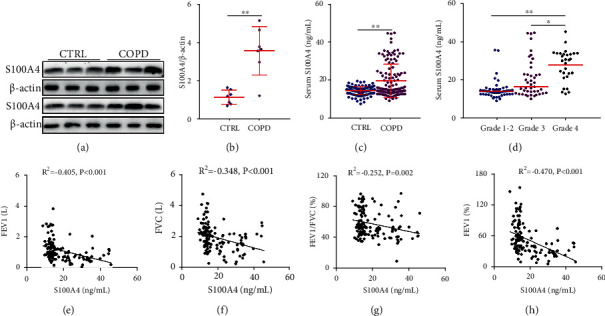
The level of S100A4 in control cases and COPD patients and the correlation of pulmonary function with serum S100A4 in COPD patients. The level of S100A4 was detected in control subjects and COPD patients. (a, b) The expression of S100A4 was measured in the lungs through western blotting between COPD patients and control subjects. (a) Representative bands were shown. (b) Quantitative analysis of scanning densitometry was tested. (c, d) The level of S100A4 was detected via ELISA. (c) The level of serum S100A4 was compared between COPD patients and control subjects (*N* = 139 for the CTRL group; *N* = 139 for the COPD group). (d) The level of serum S100A4 was compared in COPD patients with different grades. (e–h) The association of serum S100A4 and pulmonary function was evaluated through Pearson's analysis among COPD patients. (e) S100A4 vs. FEV1. (f) S100A4 vs. FVC. (g) S100A4 vs. FEV1/FVC%. (h) S100A4 vs. FEV1% (*N* = 57 for Grade 1-2 patients; *N* = 48 for Grade 3 patients; and *N* = 34 for Grade 4 patients). All data were represented as mean ± S.E.M. (*N* = 139). ^∗∗^*P* < 0.01.

The protein expression of S100A4 was detected in lung tissues of COPD patients and control cases. As shown in Figures 1(a) and 1(b), the level of pulmonary S100A4 was significantly increased in COPD patients compared with control cases. In addition, the protein expression of S100A4 in the serum was detected between COPD patients and control cases. As shown in Figure 1(c), S100A4 was increased in the serum of COPD patients. Moreover, the levels of serum S100A4 were further compared in COPD patients with different indices of pulmonary function. The results revealed that the level of serum S100A4 was higher in Grade 4 COPD patients than in Grade 1-2 and Grade 3 subjects (Figure 1(d)). Meanwhile, the levels of serum S100A4 were further compared in COPD patients with different genders. There was no difference in serum S100A4 in all COPD patients with different genders (Supplemental Figure 1(a)). Similarly, no difference was observed in COPD patients with different grades between females and males (Supplemental Figure 1(b)).

### 3.3. Correlations of Pulmonary Function with Serum S100A4 in COPD Patients

The correlation between pulmonary function and serum S100A4 was analyzed. As shown in Figure 1(e), forced expiratory volume in the first second (FEV1) was negatively correlated with serum S100A4 in COPD patients (*R*^2^ = −0.405, *P* < 0.01). Moreover, forced vital capacity (FVC) was inversely correlated with serum S100A4 in COPD patients (*R*^2^ = −0.348, *P* < 0.01) (Figure 1(f)). Besides, serum S100A4 was negatively correlated with FEV1/FVC% (*R*^2^ = −0.252, *P* = 0.002) and FEV1% (*R*^2^ = −0.470, *P* < 0.01) in COPD patients (Figures 1(g) and 1(h)). Moreover, the associations between serum S100A4 and indices of pulmonary function were evaluated through linear regression analysis among COPD patients. As shown in Table 2, univariate linear regression indicated that serum S100A4 was negatively associated with FEV1% (*β* = −0.470, 95% CI: -2.079~-1.044), FEV1/FVC% (*β* = −0.252, 95% CI: -0.810~0.156), FEV1 (*β* = −0.405, 95% CI: -0.041~-0.018), and FVC (*β* = −0.348, 95% CI: -0.048~-0.017) in COPD patients. In order to control confounding factors, age, gender, and smoking were adjusted. Multivariable linear regression found that serum S100A4 was inversely associated with FEV1% (*β* = −0.435, 95% CI: -1.962~-0.939), FEV1/FVC% (*β* = −0.212, 95% CI: -0.729~0.090), FEV1 (*β* = −0.434, 95% CI: -0.865~-0.046), and FVC (*β* = −0.405, 95% CI: -0.041~-0.018) in COPD patients (Table 2).

### 3.4. The Levels of Fibrosis and EMT between COPD Patients and Control Cases

**Figure 2:**
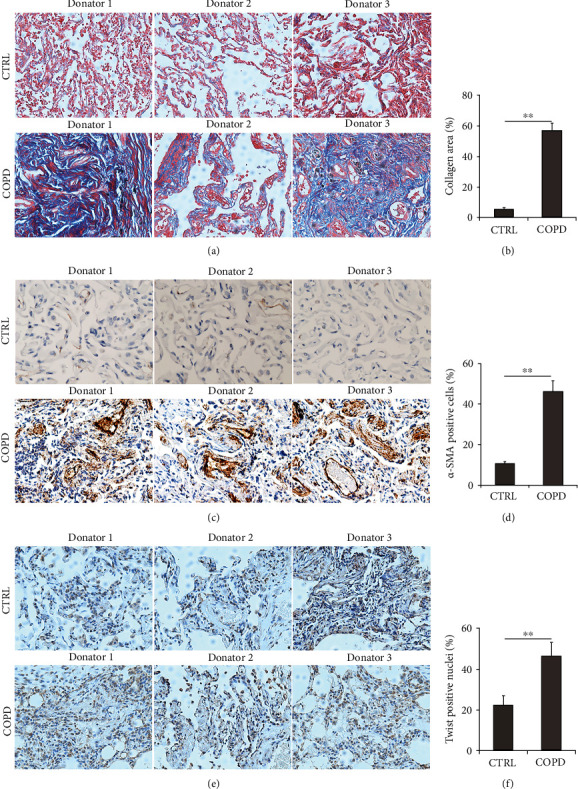
The markers of fibrosis and EMT in the lungs of COPD patients and control subjects. (a, b) The level of collagen deposition was detected via Masson's staining in the lungs between control subjects and COPD patients. (a) Representative pictures were shown. Original magnification: ×400. (b) Quantitative analysis of collagen deposition was conducted. (c, d) The level of *α*-SMA-positive cells was measured using IHC in the lungs between control subjects and COPD patients. (c) Representative pictures of *α*-SMA-positive cells were shown. Original magnification: ×400. (d) Quantitative analysis of *α*-SMA-positive cells was performed. (e, f) The level of Twist-positive nuclei was accessed using IHC in the lungs between control subjects and COPD patients. (e) Representative pictures were shown. Original magnification: ×400. (f) Quantitative analysis of Twist-positive nuclei was conducted. All data were represented as mean ± S.E.M. (*N* = 15). ^∗∗^*P* < 0.01.

The levels of pulmonary fibrosis were detected using Masson's staining between COPD patients and control cases. Masson's staining suggested that the collagen area was significantly increased in lung tissues of COPD patients than control subjects (Figures 2(a) and 2(b)). Then, *α*-smooth muscle actin (*α*-SMA), the hallmark of mature myofibroblasts, was measured through IHC between COPD patients and control cases. As shown in Figures 2(c) and 2(d), the results showed that *α*-SMA-positive cells were obviously elevated in lung tissues of COPD patients. In addition, pulmonary Twist-positive nuclei, one of the EMT transcription factors, were higher in COPD patients than in control subjects (Figures 2(e) and 2(f)).

### 3.5. The Level of S1P between COPD Patients and Control Cases

**Figure 3:**
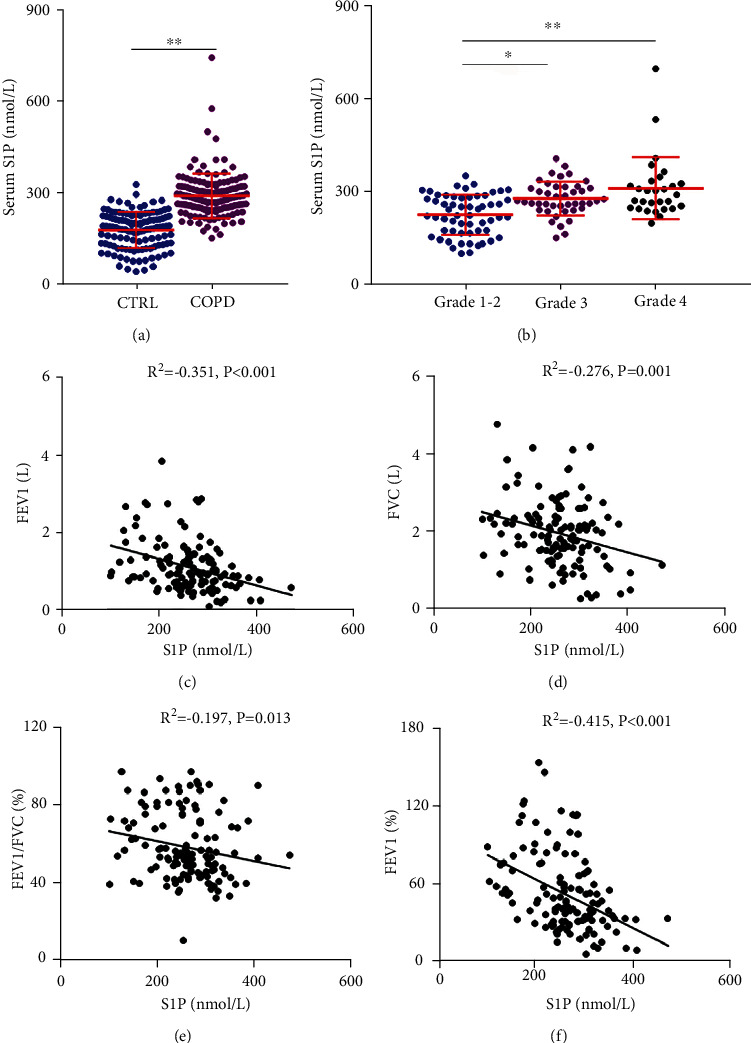
The level of serum S1P in COPD patients and control cases and the relationship between serum S1P and pulmonary function in COPD patients. The level of serum S1P was detected through ELISA. (a) The level of serum S1P was compared in control cases and COPD patients (*N* = 139 for the CTRL group; *N* = 139 for the COPD group). (b) The level of serum S1P was compared in COPD patients with different indices of pulmonary function (*N* = 57 for Grade 1-2 patients; *N* = 48 for Grade 3 patients; and *N* = 34 for Grade 4 patients). (c–f) The correlation of pulmonary function with serum S1P was evaluated via Pearson's analysis among COPD patients. (c) S1P vs. FEV1. (d) S1P vs. FVC. (e) S1P vs. FEV1/FVC%. (f) S1P vs. FEV1%. All data were represented as mean ± S.E.M. (*N* = 15). ^∗^*P* < 0.05, ^∗∗^*P* < 0.01.

Serum S1P was detected with ELISA in COPD patients and control subjects. As shown in Figure 3(a), serum S1P was significantly increased in COPD patients. Moreover, the level of serum S1P was further analyzed in COPD patients with different grades. The results showed that the level of serum S1P was lower in Grade 1-2 COPD patients than in Grade 3 and Grade 4 COPD patients (Figure 3(b)). Next, the expression of serum S1P was further compared in COPD patients with different genders. As shown in Supplemental Figure 2(a), the expression of serum S1P was similar between female COPD patients and male COPD cases. Moreover, there was no effect of gender difference on serum S1P in COPD patients with different grades (Supplemental Figure 2(b)). In addition, the correlations of serum S1P with different indices of pulmonary function were accessed among COPD patients. As shown in Figures 3(a)–3(f), serum S1P was negatively associated with FEV1 (*R*^2^ = −0.351, *P* < 0.001), FVC (*R*^2^ = −0.276, *P* = 0.001), FEV1/FVC% (*R*^2^ = −0.197, *P* = 0.013), and FEV1% (*R*^2^ = −0.415, *P* < 0.001) in COPD patients.

### 3.6. Association of S1P with S100A4 in COPD Patients and Control Cases

**Figure 4:**
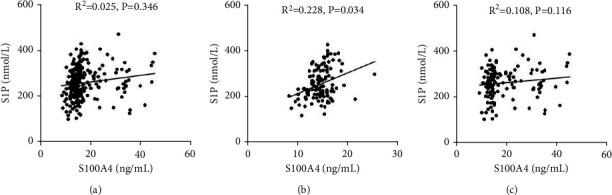
Correlation of S1P with S100A4 in the serum of COPD patients and control subjects. The correlation of S1P with S100A4 was determined through Pearson's analysis between COPD patients and control subjects. (a) The correlation of S1P with S100A4 in all subjects. (b) The correlation of S1P with S100A4 in COPD patients. (c) The correlation of S1P with S100A4 in control subjects.

The association of S1P with S100A4 in the serum of all participators was analyzed. The results revealed that there was no association between S1P and S100A4 in all participators (Figure 4(a)). Then, the association of S1P and S100A4 underwent stratified analysis in COPD patients and control cases. Though there was no association of S100A4 and S1P in control subjects (Figure 4(c)), S100A4 was positively associated with S1P in COPD patients (*R*^2^ = 0.228, *P* = 0.034) (Figure 4(b)). Then, the correlation of S100A4 and S1P was further analyzed via linear regression in all subjects. Univariate linear regression found that S100A4 was positively correlated with S1P in all cases (*β* = 0.157, 95% CI: 0.352~2.726) and COPD patients (*β* = 0.314, 95% CI: 4.311~14.518) (Table 3). After adjustment for age, gender, and smoking, multivariable linear regression revealed that S100A4 was positively correlated with S1P in COPD patients (*β* = 0.301, 95% CI: 1.112~12.365) (Table 3).

## 4. Discussion

In the present study, the levels of S100A4, S1P, and related markers of pulmonary fibrosis were detected between COPD patients and control subjects. The correlations among S100A4, S1P, and pulmonary function were analyzed in COPD patients. The present study primarily revealed the following. (1) Pulmonary S100A4 and serum S100A4 were increased in patients with COPD. (2) Serum S1P was elevated in patients with COPD. (3) The level of pulmonary fibrosis was increased in patients with COPD. (4) Serum S100A4 was positively associated with S1P and inversely associated with pulmonary function in patients with COPD.

The high incidence of COPD seriously affects the life quality of patients and brings a heavy burden to society [36]. Smoking, air pollution, genetics, and inflammation are important reasons for the development of COPD, but the pathogenesis and pathology of COPD are obscure. More and more studies have confirmed that EMT and fibrosis play central roles in the development and progression of COPD [37, 38]. S100A4 is a member of the S100 calcium-binding protein family. S100A4 has recently been discovered as a potential factor implicated in fibrotic diseases and a mesenchymal marker of EMT. Experiments in vivo and in vitro have revealed that S100A4 from fibroblasts promotes pulmonary fibrosis [9, 10]. A case-control study based on the hospital population demonstrated that serum S100A4 is elevated in patients with idiopathic pulmonary fibrosis [15]. Meanwhile, the levels of S100A4 and pulmonary fibrosis were detected in patients with COPD. The current study found that the expressions of S100A4 were elevated in COPD patients. Moreover, the higher the grade of COPD patients is, the higher the S100A4 in the serum is. Though there was no difference in serum S100A4 in COPD patients, serum S100A4 was inversely associated with pulmonary function among COPD patients. These results reveal that the expression of S100A4 is elevated in the serum and lungs of COPD patients.

Several studies from our team and other researchers have demonstrated that EMT is involved in the process of COPD [27, 28]. The epithelial markers were decreased while mesenchymal markers were increased in the lungs of COPD patients. The process of EMT can be mediated by EMT transcription factors such as Twist, Snail, and Slug. The activation of EMT transcription factors can repress E-cadherin, one epithelial marker, through binding to the E-cadherin promoter and can promote EMT [39]. In the present research, Twist-positive nuclei were obviously increased in lung tissues of COPD patients. Converging lines of evidence have identified that Twist exerts certain roles in the development of scarring and fibrosis. Alveolar epithelial cells (AECs) undergo EMT and contribute to pulmonary fibrosis [40]. Our results found that collagen deposition was increased and myofibroblasts were activated in the lungs of COPD patients. These results found that EMT and fibrosis are elevated in lung tissues of COPD patients. In addition, the role of S100A4 in EMT has been implicated in many studies [41, 42]. Recently, it has been found that S100A4 can promote pulmonary fibrosis via fibroblast activation [43]. Consequently, these data implicate that S100A4-mediated EMT and fibrosis may be involved in the occurrence and development of COPD.

S1P is a biologically active lipid synthesized intracellularly by sphingosine kinase and degraded by S1P lyase and various phosphatases. Mainly, S1P is generated in mammalian cells and released by endothelial cells, red blood cells, and monocytes [44]. S1P has different functions in several physiological and pathological conditions. In the bleomycin-induced pulmonary fibrosis mouse model, the level of S1P is elevated in the serum and lung tissues [43, 45]. Moreover, the expression of S1P is upregulated in the lung specimens and serum of pulmonary arterial hypertension rats [46, 47]. Meanwhile, several researchers have found that S1P is increased in the serum and lungs of patients with lung cancer [48, 49]. However, the correlation between S100A4 and S1P was obscure in COPD patients. In this study, serum S1P was increased in COPD patients compared with control subjects. Moreover, serum S1P was gradually elevated along with pulmonary function decline in COPD patients. These results stated that S1P elevation participated in the process of COPD. Mechanistic research has suggested that extracellular S100A4 upregulates *α*-SMA expression and collagen deposition and activates pulmonary fibroblasts through S1P [43]. Besides, S1P can directly promote EMT in pulmonary epithelial cells [50]. In the current research, correlation analysis suggested that serum S1P is inversely associated with pulmonary function among COPD patients. Although there was no obvious correlation between S100A4 and S1P in control subjects, S100A4 was positively associated with S1P in COPD patients. Further linear regression analysis indicated that S100A4 was positively associated with S1P in COPD patients. These results provide evidence that S100A4 elevation is correlated with S1P upregulation among COPD patients, indicating that S100A4 elevation may evoke EMT and fibrosis through S1P in the lungs of COPD patients.

There are several limitations to this research. First, this was a single-center research with a small sample size, and a multicenter study with a larger sample size will further prove these results. Second, this is only a case-control study, and the causal link between the elevation of S100A4 and S1P and the decline of pulmonary function was unclear. Further animal experiments may clarify this problem. Third, the mechanism of S1P-mediated EMT was unclear in this case-control study, and only experiments in vivo and in vitro can resolve this confusion.

## 5. Conclusions

In summary, this research primarily evaluated the associations among S100A4, S1P, and pulmonary function in patients with COPD based on a case-control study. Our results revealed that S100A4 is increased in the lungs and serum of COPD patients. Meanwhile, the markers of fibrosis and EMT are elevated in the lungs of COPD patients. Moreover, serum S1P has risen in COPD patients. Correlative analysis revealed that pulmonary function is inversely associated with S1P and S100A4 in COPD patients. Additionally, serum S1P is positively associated with S100A4 in COPD patients. These results provide evidence that the elevation of S100A4 and S1P may be involved in the onset and progression of COPD patients.

## Figures and Tables

**Table 1:** Demographic information and clinical characteristics.

Variable	CTRL (*N* = 139)	COPD (*N* = 139)	*P*
Age (years)	72.35 ± 5.63	73.84 ± 0.69	0.201
Male, *N* (%)	100 (71.9)	103 (74.1)	0.394
Ex-smoker, *N* (%)	42 (30.2)	113 (75.3)	<0.001
WBC (10^9^/L)	5.90 (5.37, 6.81)	6.80 (5.11, 8.90)	<0.05
Neutrophil (10^9^/L)	3.08 (2.58, 3.88)	4.61 (3.28, 6.72)	<0.05
Lymphocyte (10^9^/L)	2.17 (1.88, 2.59)	1.20 (0.82, 1.49)	<0.01
Eosinophil (10^9^/L)	0.13 (0.07, 0.18)	0.11 (0.03, 0.19)	0.365
Monocyte (10^9^/L)	0.41 (0.33, 0.50)	0.56 (0.38, 0.75)	0.071
Basophil (10^9^/L)	0.01 (0.01, 0.02)	0.02 (0.01, 0.03)	0.541
FEV1 (%)	N.A.	44.5 (30.7, 68.5)	N.A.
FEV1/FVC (%)	N.A.	53.3 (44.1, 71.5)	N.A.
FEV1 (L)	N.A.	0.94 (0.66, 1.35)	N.A.
FVC (L)	N.A.	1.90 (1.40, 2.31)	N.A.
CRP (*μ*g/mL)	3.2 (1.6, 16.9)	8.8 (2.2, 54.9)	<0.01
IL-6 (pg/mL)	2.5 (1.0, 10.5)	6.0 (2.0, 21.3)	<0.01

WBC: white blood cell; FEV1: forced expiratory volume in one second; FVC: forced vital capacity; IL-6: interleukin-6; CRP: C-reactive protein; N.A.: not available.

**Table 2:** Association of serum S100A4 with pulmonary function in COPD patients.

Variables	Univariate, *β* (95% CI)	*P*	Multivariable, *β* (95% CI)	*P*
FEV1 (%)	-0.470 (-2.079, -1.044)	<0.001	-0.435 (-1.962, -0.939)	<0.001
FEV1/FVC (%)	-0.252 (-0.810, 0.156)	0.004	-0.212 (-0.729, 0.090)	0.013
FEV1 (L)	-0.405 (-0.041, -0.018)	<0.001	-0.434 (-0.865, -0.046)	<0.001
FVC (L)	-0.348 (-0.048, -0.017)	<0.001	-0.405 (-0.041, -0.018)	<0.001

^∗^Age, gender, and smoking were adjusted.

**Table 3:** Association of serum S100A4 with S1P in all subjects.

Variables	Univariate, *β* (95% CI)	*P*	Multivariable, *β* (95% CI)	*P*
All cases	0.157 (0.352, 2.726)	0.011	0.162 (-0.094, 2.565)	0.068
COPD group	0.314 (4.311, 14.518)	<0.001	0.301 (1.112, 12.365)	0.034
CTRL group	0.149 (-0.147, 2.406)	0.082	0.123 (-0.059, 3.354)	0.087

^∗^Age, gender, and smoking were adjusted.

## Data Availability

The data used to support the findings of this study are available from the corresponding authors upon request.

## Supplementary Materials

Supplementary 1 Supplemental Figure 1: the level of serum S100A4 in COPD patients with different genders. The level of serum S100A4 detected through ELISA in COPD patients with different genders. (a) The level of serum S100A4 was detected in male and female COPD patients. (b) The level of serum S100A4 was detected in female and male COPD patients with different grades. Supplemental Figure 2: the level of serum S1P in COPD patients with different genders. The level of serum S1P detected via ELISA in COPD patients with different genders. (a) The level of serum S1P was detected in male and female COPD patients. (b) The level of serum S1P was detected in female and male COPD patients with different grades.

Supplementary 2 Supplemental Table 1: demographic information and clinical characteristics.
