# Supplementary material for: Associations among S100A4, Sphingosine-1-Phosphate, and Pulmonary Function in Patients with Chronic Obstructive Pulmonary Disease
Source: Oxid Med Cell Longev. 2022 Feb 3;2022:6041471. doi: 10.1155/2022/6041471 (PMC8837900; doi:10.1155/2022/6041471)
Supplement: Supplementary 1 — Supplemental Figure 1: the level of serum S100A4 in COPD patients with different genders. The level of serum S100A4 detected through ELISA in COPD patients with different genders. (a) The level of serum S100A4 was detected in male and female COPD patients. (b) The level of serum S100A4 was detected in female and male COPD patients with different grades. Supplemental Figure 2: the level of serum S1P in COPD patients with different genders. The level of serum S1P detected via ELISA in COPD patients with different genders. (a) The level of serum S1P was detected in male and female COPD patients. (b) The level of serum S1P was detected in female and male COPD patients with different grades. [file 6041471.f1.doc]

**Supplementary Data**


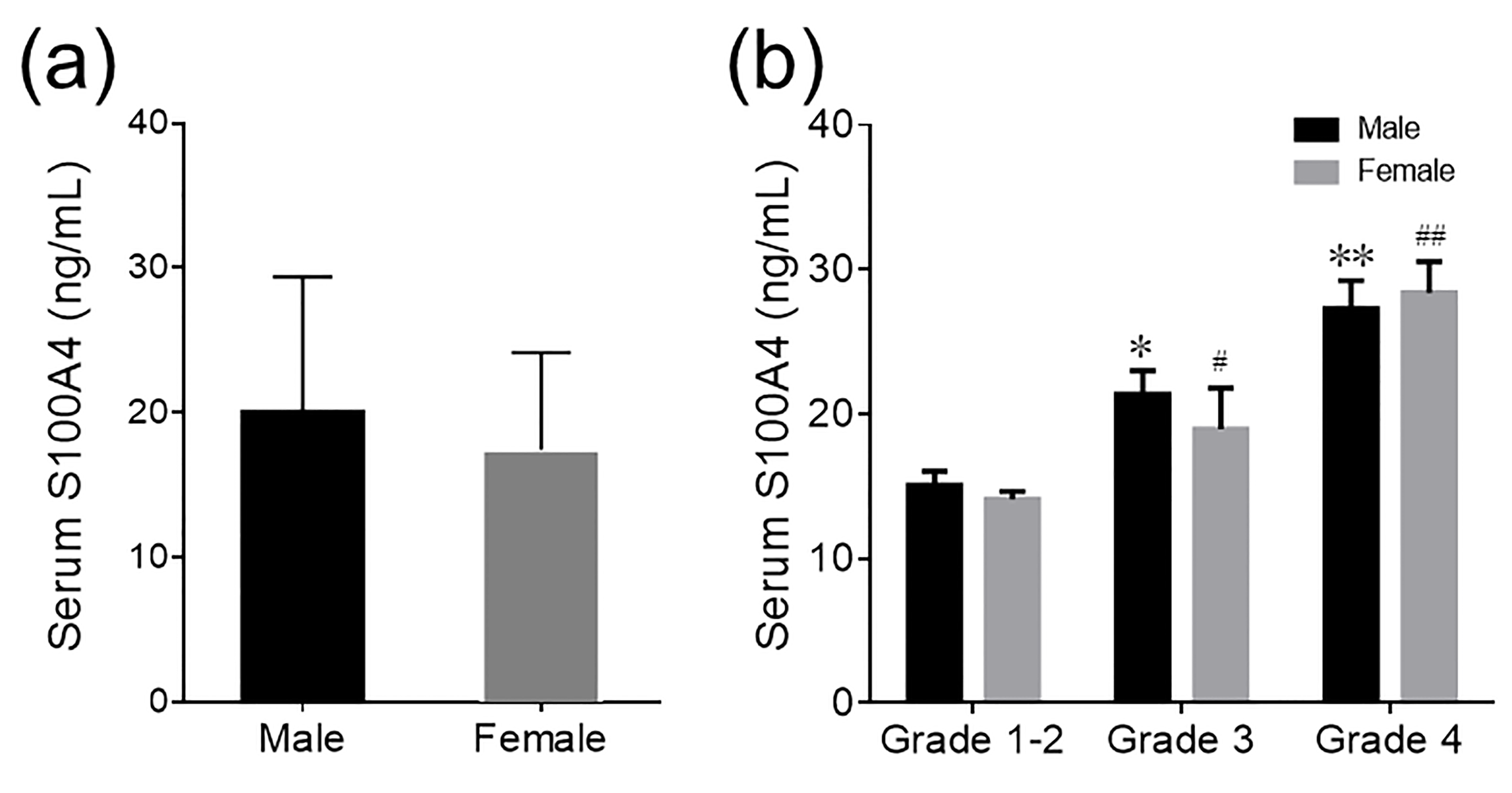
**Supplemental Figure 1. The level of serum S100A4 in COPD patients with different gender.** The level of serum S100A4 detected through ELISA in COPD patients with different gender. (a) The level of serum S100A4 was detected in male and female COPD patients. (b) The level of serum S100A4 was detected in female and male COPD patients with different grades.


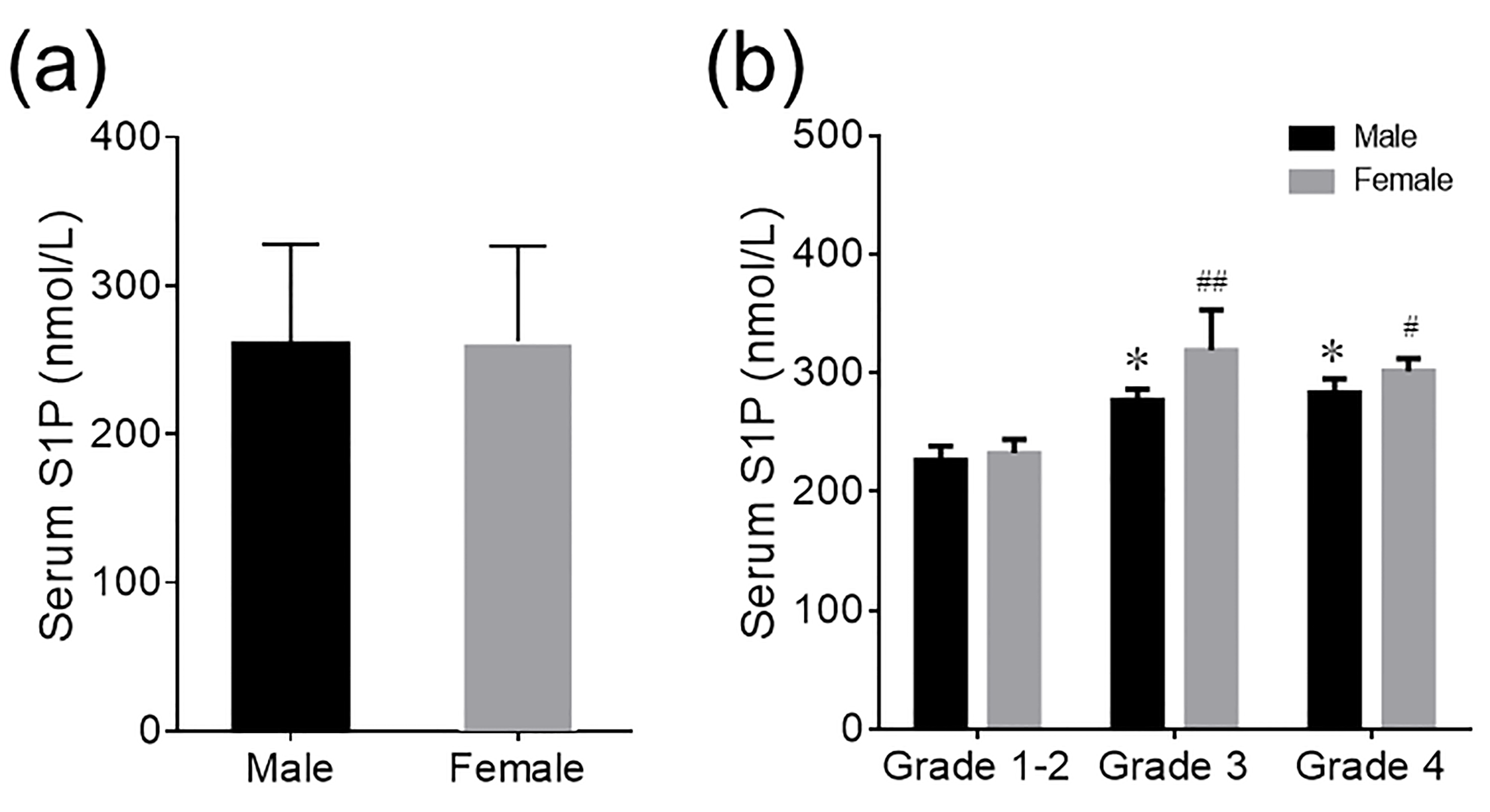


**Supplemental Figure 2. The level of serum S1P in COPD patients with different gender.** The level of serum S1P detected via ELISA in COPD patients with different gender. (a) The level of serum S1P was detected in male and female COPD patients. (b) The level of serum S1P was detected in female and male COPD patients with different grades.
