# Supplementary material for: Associations among S100A4, Sphingosine-1-Phosphate, and Pulmonary Function in Patients with Chronic Obstructive Pulmonary Disease
Source: Oxid Med Cell Longev. 2022 Feb 3;2022:6041471. doi: 10.1155/2022/6041471 (PMC8837900; doi:10.1155/2022/6041471)
Supplement: Supplementary 2 — Supplemental Table 1: demographic information and clinical characteristics. [file 6041471.f2.doc]

Supplemental Table 1. Demographic information and clinical characteristics

| Variables | CTRL | |  | COPD | |  |
| --- | --- | --- | --- | --- | --- | --- |
|  | Male (n=100) | Female (n=39) | *P* | Male (n=103) | Female (n=36) | *P* |
| Age (years) | 71.68±1.62 | 72.48±1.18 | 0.707 | 74.34±0.76 | 72.31±1.55 | 0.326 |
| Ex-smoker, n (%) | 36 (36.0) | 6 (15.4) | 0.017 | 64 (62.1) ** | 3 (8.33) | ＜0.001 |
| WBC (10^9^/L) | 6.02 (5.38, 6.82) | 5.98 (5.10, 7.07) | 0.789 | 6.84 (5.27, 9.30) * | 6.59 (4.97, 8.62) ^#^ | 0.329 |
| Neutrophil (10^9^/L) | 3.21 (2.54, 3.82) | 3.40 (2.71, 4.17) | 0.280 | 4.60 (3.40, 6.90) ** | 4.61 (3.05, 5.47) ^##^ | 0.313 |
| Lymphocyte (10^9^/L) | 2.18 (1.92, 2.58) | 2.13 (1.73, 2.69) | 0.374 | 1.13 (0.82, 1.49) ** | 1.34 (0.85, 1.60) ^##^ | 0.225 |
| Eosinophil (10^9^/L) | 0.15 (0.07, 0.20) | 0.09 (0.06, 0.14) | 0.008 | 0.10 (0.02, 0.19) | 0.11 (0.05, 0.20) | 0.692 |
| Monocyte (10^9^/L) | 0.44 (0.35, 0.51) | 0.34 (0.30, 0.45) | 0.017 | 0.58 (0.40, 0.76) | 0.51 (0.33, 0.71) | 0.187 |
| Basophil (10^9^/L) | 0.02 (0.01, 0.03) | 0.02 (0.01, 0.03) | 0.465 | 0.02 (0.01, 0.04) | 0.02 (0.01, 0.03) | 0.426 |
| FEV1 (%) | N.A. | N.A. | N.A. | 41.50 (30.60, 57.08) | 70.40 (32.63, 105.25) | 0.011 |
| FEV1/FVC (%) | N.A. | N.A. | N.A. | 51.48 (43.55, 62.74) | 68.66 (53.68, 80.06) | ＜0.001 |
| FEV1 (L) | N.A. | N.A. | N.A. | 0.94 (0.70, 1.32) | 1.01 (0.53, 1.62) | 0.943 |
| FVC (L) | N.A. | N.A. | N.A. | 2.04 (1.50, 2.50) | 1.49 (1.06, 2.04) | 0.007 |
| CRP (μg/mL) | 3.1 (1.3, 14.6) | 2.6 (1.2, 15.8) | 0.121 | 11.10 (3.10, 63.08) ** | 3.45 (0.85, 11.00) ^##^ | 0.009 |
| IL-6 (pg/mL) | 3.0 (1.1, 9.6) | 2.7 (1.0, 10.4) | 0.254 | 6.40 (2.10, 24.60) ** | 5.25 (1.15, 14.95) ^##^ | 0.318 |

WBC: White blood cell; FEV1: Forced expiratory volume in one second; FVC: Forced vital capacity; IL-6: Interleukin-6; CRP: C-reactive protein; N.A., Not available.

**P*＜0.05, ***P*＜0.01 as compared with Male subjects. ^#^*P*＜0.05, ^##^*P*＜0.01 as compared with Female subjects.
